# Developmental Transcriptomic Features of the Carcinogenic Liver Fluke, *Clonorchis sinensis*

**DOI:** 10.1371/journal.pntd.0001208

**Published:** 2011-06-28

**Authors:** Won Gi Yoo, Dae-Won Kim, Jung-Won Ju, Pyo Yun Cho, Tae Im Kim, Shin-Hyeong Cho, Sang-Haeng Choi, Hong-Seog Park, Tong-Soo Kim, Sung-Jong Hong

**Affiliations:** 1 Department of Medical Environmental Biology and Research Center for Biomolecules and Biosystems, Chung-Ang University College of Medicine, Seoul, Republic of Korea; 2 Division of Malaria and Parasitic Diseases, National Institute of Health, Korea Centers for Disease Control and Prevention, Osong, Chungbuk, Republic of Korea; 3 Department of Infection Biology, Zoonosis Research Center, Wonkwang University School of Medicine, Chonbuk, Republic of Korea; 4 Genome Research Center, Korea Research Institute of Bioscience and Biotechnology and University of Science and Technology, Daejeon, Republic of Korea; 5 Department of Parasitology, Inha University School of Medicine, Incheon, Republic of Korea; Khon Kaen University, Thailand

## Abstract

*Clonorchis sinensis* is the causative agent of the life-threatening disease endemic to China, Korea, and Vietnam. It is estimated that about 15 million people are infected with this fluke. *C. sinensis* provokes inflammation, epithelial hyperplasia, and periductal fibrosis in bile ducts, and may cause cholangiocarcinoma in chronically infected individuals. Accumulation of a large amount of biological information about the adult stage of this liver fluke in recent years has advanced our understanding of the pathological interplay between this parasite and its hosts. However, no developmental gene expression profiles of *C. sinensis* have been published. In this study, we generated gene expression profiles of three developmental stages of *C. sinensis* by analyzing expressed sequence tags (ESTs). Complementary DNA libraries were constructed from the adult, metacercaria, and egg developmental stages of *C. sinensis*. A total of 52,745 ESTs were generated and assembled into 12,830 *C. sinensis* assembled EST sequences, and then these assemblies were further categorized into groups according to biological functions and developmental stages. Most of the genes that were differentially expressed in the different stages were consistent with the biological and physical features of the particular developmental stage; high energy metabolism, motility and reproduction genes were differentially expressed in adults, minimal metabolism and final host adaptation genes were differentially expressed in metacercariae, and embryonic genes were differentially expressed in eggs. The higher expression of glucose transporters, proteases, and antioxidant enzymes in the adults accounts for active uptake of nutrients and defense against host immune attacks. The types of ion channels present in *C. sinensis* are consistent with its parasitic nature and phylogenetic placement in the tree of life. We anticipate that the transcriptomic information on essential regulators of development, bile chemotaxis, and physico-metabolic pathways in *C. sinensis* that presented in this study will guide further studies to identify novel drug targets and diagnostic antigens.

## Author Summary

*Clonorchis sinensis* is a significant pathogen that causes clonorchiasis, which is endemic to East Asian countries. This fluke provokes acute inflammation and chronic hyperplasic changes in the biliary tracts. *C. sinensis* promotes cholangiocarcinoma, and has been classified as a Group 1 biological carcinogen, alongside *Opisthorchis viverrini*, by the World Health Organization. Recently, transcriptomes for adult liver flukes have been reported with the molecular functionalities predicted on the bases of their transcriptomic data sets. We generated the developmental *C. sinensis* transcriptome for three different developmental stages, revealing that most functional genes were differentially expressed in each developmental stage; only a small proportion of the expressed genes were shared between the three stages. The developmental transcriptome describes the gene expression landscapes of *C. sinensis* adults, metacercariae, and eggs, and provides insight into how this fluke adapts to the distinctly different environments provided by its various hosts. We anticipate that the transcriptome will contribute significantly to the identification of intervention points along the developmental stages and allow the exploitation of novel potential targets for diagnostic, drug, and vaccine development purposes.

## Introduction

*Clonorchis sinensis* causes clonorchiasis, which is endemic to Korea, China, Taiwan and Vietnam; approximately 15 million people are estimated to be infected [1]–[3]. *C. sinensis* is a significant pathogen both from an epidemiological and clinical perspective, as people who develop clonorchiasis are debilitated, thereby negatively impacting socio-economic activities. In *C. sinensis* endemic areas, inhabitants become infected by eating raw or inappropriately cooked fresh water fish caught from water bodies near their villages [4]. Fresh water fish are the hosts of *C. sinensis* metacercariae, which is the infective stage to humans.

Once *C. sinensis* eggs reach a fresh water body, they develop into miracidiae. When ingested by freshwater snails, the miracidia escapes from the egg and transforms into sporocysts, and then into rediae within several weeks. The cercariae emerge into fresh water and swim in search of freshwater fish, the second intermediate host. The cercaria penetrates the skin of a freshwater fish and its body becomes encysted by a cyst wall, followed by transformation into a metacercaria. Almost all freshwater fish can serve as the second intermediate hosts, with the highest infection rate and metacercarial burden found in the topmouth gudgeon, *Pseudorasbora parva*. When ingested by humans and other mammals, the metacercariae are retained for a while in the stomach and then passed down to the duodenum. The metacercariae excyst there and the hatched, juvenile *C. sinensis* migrate up into the bile duct. The juvenile flukes grow to adults that produce eggs in the biliary passages of the mammalian host.

C. *sinensis* flukes in the biliary tracts lacerate and apply pressure to the epithelia, and excrete waste products from their excretory bladder and regurgitate residual digests from their intestinal ceca. In addition, ovigerous *C. sinensis* adults excrete uterine fluid with high protein content when they ovulate. These various excretory and secretory products act as chemical irritants that provoke inflammation, epithelial hyperplasia, and periductal fibrosis in the biliary tracts. In human clonorchiasis patients, frequent symptoms are epigastric discomfort and dull pain, mild fever, loss of appetite, diarrhea, and jaundice [5]. Moreover, clonorchiasis has epidemiologically been reported to be associated with cholangiocarcinoma [6]–[8]. Furthermore, experimental studies have shown that *C. sinensis* infection induces the differentiation of liver oval cells into a bile duct cell lineage and promotes the development of cholangiocarcinoma in golden hamsters [9], [10]. Recently, *C. sinensis* was officially classified along with *Opisthorchis viverrini* as a Group 1 biological carcinogen by the World Health Organization [11]. Among the excretory-secretory products produced by liver flukes, granulin was identified as a mitogenic agent capable of stimulating cell proliferation and epithelial hyperplasia [12]. By binding to Toll-like receptors, the excretory-secretory products of adult flukes activate the NF-kB pathway resulting in increased expression of the pro-inflammatory cytokine, IL-6 [13], which in turn leads to the production of reactive oxygen radicals. The endogenous reactive radicals damage DNA and could initiate carcinogenesis [14].

Expressed sequence tags (ESTs) generated from cDNA libraries cover a large proportion of functional mRNAs and can be assembled into overlapping contigs coding for almost complete open reading frames [15]. *Schistosoma mansoni* and *S. japonicum* were the first flukes for which transcriptome data was published [16], [17]; these studies stimulated the generation of ESTs and functional cataloging of these ESTs from the human-infecting liver flukes, *C. sinensis*, *O. viverrini*, and *Fasciola hepatica* [18]–[22]. The recent widespread availability of next-generation sequencing technology has also stimulated high-throughput analyses of the transcriptomes of liver flukes with a focus on the pathobiological characteristics of these adult liver fluke transcriptomes [23], [24]. Given that *C. sinensis* adults are considered carcinogenic agents, they are predicted to express genes encoding proteins that are also known to be involved in cancer development [25].

To further elucidate the pathogenesis and carcinogenesis provoked by *C. sinensis* infection, comprehensive molecular and genetic information covering the different developmental stages of this parasite is required. In this study, we generated and sequenced transcriptome-scale ESTs from three developmental stages of *C. sinensis* and investigated the biological properties, growth, host adaptations, and pathogenic features of these different developmental stages.

## Materials and Methods

### Ethics statement

Rabbits were handled in an accredited Korea Food and Drug Administration animal facility in accordance with the AAALAC International Animal Care policies (Accredited Unit, Korea FDA; Unit Number 000996). Approval for animal experiments was obtained from Korea FDA animal facility (NIH-06-15, NIH-07-16 and NIH-08-19).

### Parasite resources, culture and RNA extraction

*C. sinensis* metacercariae were collected from naturally infected *P. parva* caught in Jinju, Korea, and Shenyang, China. The fish were ground and digested artificially in gastric juice for 1 hr at 37°C. Particulate material was filtered out using a sieve with 0.15 mm mesh and washed several times with 0.85% saline. *C. sinensis* metacercariae were identified and collected under a dissecting microscope. Male New Zealand White rabbits, 1.5–3.0 kg (Samtaco Inc., Korea) were infected with 500 metacercariae each, and adult flukes were recovered from the bile ducts of these experimental rabbits 2 months after the infection. Bile juice collected from *C. sinensis*-infected rabbits was centrifuged at 2,000 *g* for 10 min and *C. sinensis* eggs were collected from the sediment. To extract total RNA, adult flukes, metacercariae, and eggs of *C. sinensis* were put into liquid nitrogen in a pre-chilled mortar on dry ice and pulverized using a Mixer Mill MM301 (Retsch GmbH, Haan, Germany). Total RNA was extracted from the ground tissues using TRI reagent (MRC, Inc., Cincinnati, OH, USA). Poly(A^+^) mRNA was selected from the total RNA using the Absolutely mRNA Purification Kit (Stratagene, La Jolla, CA, USA) according to the manufacturer's instruction. The amounts of total RNA and mRNA were determined by measuring the absorbance at 260 nm and the degree of protein contamination was assessed by calculating the ratio of the absorbance at 260 nm to that at 280 nm. RNA integrity was assessed by examining ribosomal RNA bands on 1% RNA agarose gels stained with ethidium bromide.

### Construction of cDNA libraries

Using the poly(A^+^) mRNAs generated as described above, cDNA libraries of the three developmental stages of *C. sinensis* (adult, metacercaria, and egg) were constructed using the directional λ ZAP cDNA synthesis/Gigapack Ш Gold cloning kit (Stratagene, La Jolla, CA, USA). First stand cDNAs were synthesized from mRNAs primed at the poly-A tail using reverse transcriptase and an oligo-dT linker-primer containing an *Xho*I restriction enzyme site. Following second strand synthesis, an *EcoR* I linker was ligated to the 5′-termini followed by digestion with the restriction enzyme *Xho*I. These synthesized and assembled double strand cDNAs were size-fractionated using Sepharose® CL-2B gel filtration column chromatography. cDNA fractions longer than 500 bp were ligated into the ZAP Express vector pBK-CMV and the ligation products were packaged *in vitro* into cDNA libraries using the ZAP Express cDNA Gigapack Ш Gold cloning Kit (Stratagene). cDNAs were directionally cloned into the pBK-CMV vector, which allows both prokaryotic and eukaryotic expression of large sequences and *in vivo* excision into a phagemid vector. The adult, metacercaria, and egg cDNA libraries were plated onto LB-kanamycin plates, 23.5 cm×23.5 cm, coated with X-gal/IPTG for blue/white selection. White colonies were randomly picked and inoculated into each well of a 384-well plate (Corning Co., Cortland, NY, USA) containing 40 µl Terrific Broth/kanamycin, followed by incubation for 16 hr at 37°C. For storage, the culture media in the 384-well plates were mixed with an equal volume of glycerol solution (65% glycerin, 0.1 M MgSO4, 0.025 M Tris-HCl, pH 8.0) and stored at −80°C. To assess cDNA quality, additional cDNA libraries were constructed in the pBluescript SK(+) vector for the adult and in the pAD-GAL42.1 vector for the metacercaria.

### cDNA sequencing

A total of 60,768 colonies were picked: 30,144 from the adult cDNA library, 20,256 from the metacercaria cDNA library, and 10,368 from the egg cDNA library. Single plasmid colonies were transferred into 540 µl of Terrific Broth medium supplemented with 50 µg/ml kanamycin in a 96-deep well plate and incubated at 37°C overnight with gentle rotation (550 rpm). Plasmids containing *C. sinensis* cDNA were extracted using an alkaline lysis method [26], [27]. The sequences of the cloned *C. sinensis* cDNAs were determined using the BigDye Terminator Cycle Sequencing Kit, ver. 3.1 (Applied Biosystems, Foster City, CA, USA). Sequencing reactions were performed in a 3-µl volume containing 250 ng plasmid DNA, 0.5 pmole universal primer, 0.87 µl of 5X Sequencing buffer, and 1.38 µl of distilled water. The cycling profile consisted of 35 cycles of denaturation at 96°C for 10 seconds, annealing at 50°C for 5 seconds, and extension at 60°C for 4 min. Sequencing products were purified via ethanol precipitation and read on an ABI 3730XL DNA Analyzer (Applied Biosystems, Foster City, CA). The T3 forward primer and T7 reverse primer were used as sequencing primers.

### DNA sequence trimming and assembly

Nucleotide sequences of the 60,768 clones were read once from the 5′-end. The vector and adapter sequences were trimmed off all reads, as well as nucleotide stretches with a Phred score of 20 or less and poly A/T stretches [28], [29]. Reads shorter than 100 bp were then filtered out of the analyses. A total of 52,745 reads that survived these quality control filters were assembled into clusters using the TGICL and CAP3 programs with the following parameters: an offset of 40 bp overlap, 95% minimum identity, and a maximum mismatched overhang of 30 bp [30], [31]. Nucleotide sequences of the reads reported in this paper are registered in the DDBJ/EMBL/GenBank databases under the accession numbers FS126466-FS179210.

### Bioinformatic analyses of ESTs

To annotate the assembled EST clusters, the nucleotide sequences of the clusters were translated into putative polypeptide sequences and these sequences were blasted against the NCBI non-redundant nucleotide and protein databases using the parameters of more than 30 matched amino acids, an identity greater than 25%, and an E-value less than 1e^−4^ using BLASTX. The domain structures of the translated polypeptides were predicted using InterProScan (data version v14.0) and an E-value of less than 1e^−4^ [32] and their potential function was assessed by gene ontology (GO) analysis. Moreover, we manually curated the gene descriptions in the databases by selecting known genes with a significant E-value to prevent incorrect assignment of annotated genes. To further enhance the reliability of the data and provide more accurate gene predictions for *C. sinensis*, we chose the species closest to *C. sinensis* for which data were available.

Signal peptides in the EST clusters were searched and categorized based on their function. The open reading frame of a conceptual polypeptide was first translated from the cluster sequence using OrfPredictor [33] (https://fungalgenome.concordia.ca/tools/OrfPredictor.html). The signal peptide sequences of the conceptual polypeptides were predicted using the SignalP 3.0 server [34] (http://www.cbs.dtu.dk/services/SignalP) and the subcellular localizations of proteins were analyzed using PSORTb [35] (http://psort.nibb.ac.jp). Secondary structure features of the peptides such as alpha helices and intervening loops were predicted using TMHMM version 2.0 [36] (http://www.cbs.dtu.dk/services/TMHMM/). Putative B-cell epitopes in *C. sinensis* EST-encoding proteins were predicted using the ABCpred database [37] (http://www.imtech.res.in/raghava/abcpred/ABC_submission.html).

To generate developmental gene expression profiles of *C. sinensis*, the number of ESTs in each contig was counted according to developmental stage and analyzed using Fisher's exact test using a significance level of *P*<0.01 at IDEG6 [38] (http://telethon.bio.unipd.it/bioinfo/IDEG6_form/). Putative SNPs in the EST sequences were determined using the AutoSNP program [39].

To gain insight into the evolutionary history of *C. sinensis* and to investigate parasitism-related genes, the global similarity of *C. sinensis* whole ESTs at the amino acid sequence level were compared to those of other parasites and free-living platyhelminthes using the SimiTri program (cut-off score: 50) [40]. Whole EST sequences of comparator organisms were retrieved from the NCBI protein database. The relative similarities of the gene sequences of *C. sinensis* to those of other species were analyzed using TBLASTX and the SimiTri viewer. Bulk sequences of the comparator species were downloaded from the GenBank EST databases. Sequences with BLAST scores (bit score values) higher than 50 were collected from each large dataset. Nucleotide versus nucleotide comparisons of the dataset of interest to the different databases were performed using the TBLASTX algorithm. The primary data consisted of the similarity values of each sequence from the chosen database. The primary data was transformed into input for the SimiTri viewer. A gene is indicated as a square tile, and these tiles are colored by similarity scores to other datasets. Genes that were similar to genes in only one other database are not shown. Genes that showed similarity to genes in only two databases are shown as lines joining the two databases.

### URL

More detailed information and raw data are accessible at http://grc.kribb.re.kr/pipeline2/.

### ID of genes

The ID numbers for genes and proteins mentioned in the text refer to tables. The others are as follow: K^+^-channel, CL3811; Ca^+2^-channels, CL6309, CSM01492; Na^+^-Channel, CSA10278; Cl^−^ channel, CL2019; Sodium transporters, CL5256, CL25, CL6319, CL420, CSA00901, CSA10105, CSA10217, CSA19634; Na+/K+-ATPases, CL2552, CSA23629; Glucose transporters, CL272, CL25; Amino acid transporters, CL1075, CL1111; Zinc transporter, CL1482; Phosphate transporters, CL5278, CSA12737; Fatty acid transporter, CL384. Apoptosis, CL632, CL635, CL621; Cell proliferation, CL1924, CL134, CL6028, CL894; cancer development, CL644, CL557, CL3820, CL1644, CL1801, CL5519, CL5976, CL1379, CL2881, CL2147, CL684, CL894, CL25, CL1289, CL1087, CL545, CL1679, CL421, CL111; Neuroreceptors, CL1876, CSM05821, CSM11397; Neurotransmitter-related proteins, CL4260, CSA10291, CL1504, CL5536, CSM14836, CL5972.

## Results and Discussion

### The *C. sinensis* transcriptome

To generate transcriptomes and evaluate developmental gene expression in *C. sinensis*, 60,768 clones were selected randomly from cDNA libraries of the adult, metacercaria, and egg developmental stages. After stringent quality filtering through base calling, vector sequence trimming, repeat masking, and contaminant screening, 52,745 high quality reads remained (success index of 86.8%) consisting of 27,070 reads from the adult stage, 15,872 reads from the metacercaria stage, and 9,803 reads from the egg stage **(** **Table 1** **)**. The high quality reads were assembled and clustered into 12,830 *C. sinensis* assembled EST sequences (CsAEs) comprising 7,184 contigs and 5,646 singletons [31]. The length of the CsAEs ranged from 100–3,328 bp with an average length of 724 bp. Over 50% of the CsAEs were between 500–799 bp. More than 70% of the CsAEs consisted of less than 30 EST members, while the largest single CsAE had 643 EST members.

**Table 1:** Transcriptome feature of three developmental stages from *Clonorchis sinensis.*

	Adults	Metacercariae	Eggs	All
**Total sequence reads**	30,144	20,256	10,368	60,768
**Total analyzed reads** +	27,070	15,872	9,803	52,745
**Average EST size (bp after trimming)**	528	524	569	522
**Total number of assembled sequences**	7,779	5,398	2,660	12,830‡
**Contigs**	3,921	2,728	1,523	7,184
**Singlets**	3,858	2,670	1,137	5,646
**Average contigs size (bp)**	720	671	758	724
**Total known genes** *	3,993	2,718	1,630	6,413
**Unigene number**	3,488	2,427	1,504	5,269
**Unigenes matched to ** ***S. mansoni*** ** or S. ** ***japonicum***	1,674(48%)	1,138(47%)	868(58%)	2,356(45%)
**Unigenes matched to other organisms**	1,814(52%)	1,289(53%)	636(42%)	2,913(55%)
**Total unknown genes**	3,786	2,680	1,030	6,417

**+:** : Total analyzed reads were determined using the stringent quality filtering and contained reads with a minimum of 100 bases and a phred quality≥20.

*: Total known genes were determined by homology ≥25% and length of minimum exact match ≥30 amino acids by using BLASTX homology search.

**‡:** : Each stage (column) was generated from its own dataset. Independently, data of all stage were assembled from the combination of adults, metacercariae and eggs.

### Developmental gene expression

A total of 52,745 reads collected from the adult, metacercaria, and egg stages were assembled into 7,184 contigs. Of these contigs, 1,887 (26.3%) were shared by two developmental stages; 648 contigs between the adult and egg stages, 974 between the adult and metacercaria stages, and 261 between the metacercaria and egg stages. A small portion of the transcriptome (564 contigs; 7.9%) occurred in all three developmental stages, suggesting that some of these are housekeeping genes expressed constitutively across the life stages of *C. sinensis*. A large number of the contigs (4,733; 65.9%) occurred in one of the three developmental stages **(Figure S1)**. This finding suggests that genes associated with growth can be identified from the *C. sinensis* developmental transcriptome. The expression levels of these genes, as determined by the number of ESTs contained within a cluster, were compared to investigate stage-specific patterns of transcription based on an arbitrary cut-off as well as the statistical significance of the expression differences. In *C. sinensis*, 119 contigs in the adult stage, 48 in the metacercaria stage, and 134 in the egg stage were significantly differentially expressed. The majority of CsAEs obtained from each developmental stage were non-annotated or hypothetical transcripts. The unknown CsAEs were broadly distributed at each stage, indicating that *C. sinensis* may have a unique developmental mechanism compared to other parasites.

### Functional annotation

Our manual curation of the search reports generated by BLASTX and InterPro increased the annotation rate and accuracy of the functional notations of the CsAEs. Of the 12,830 CsAEs, 7,132 (55.6%) were found to have significant sequence similarities to sequences in the NCBI NR database and/or in InterPro [41], while the remaining 5,698 (44.4%) CsAEs had no homolog **(Figure S2).** One-half of the proteins translated from the CsAEs were annotated by BLASTX and found to be similar to proteins found in *Schistosoma japonicum*. Among them, the sequences of hsp90, RNA binding protein, and actin were highly conserved between *C. sinensis* and *S. japonicum*. Another 44% of the CsAEs were unique gene transcripts with no homolog in the databases searched.

### Gene ontology

**Figure 1:**
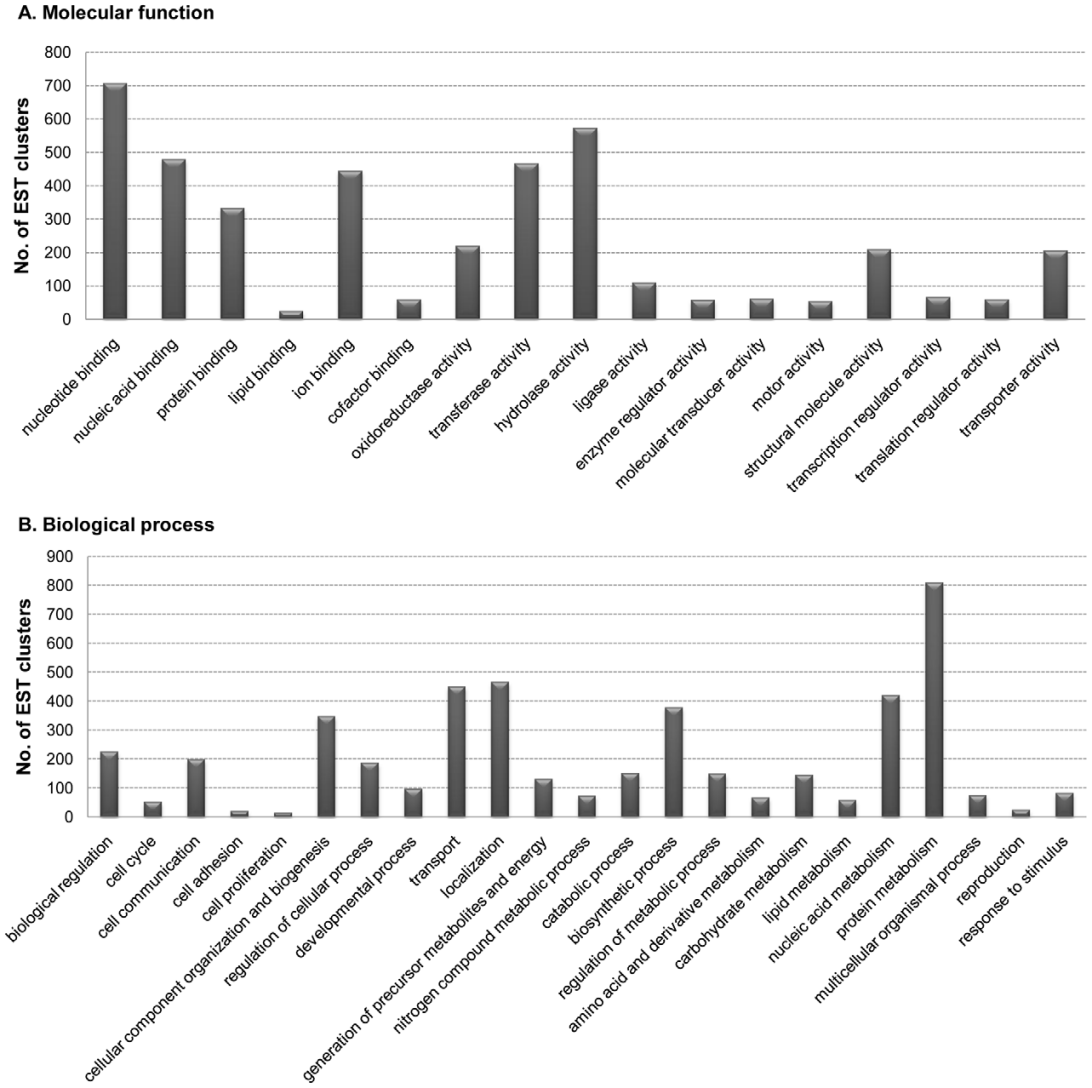
Predicted functions of *C. sinensis* transcripts based on gene ontology analysis. Distribution of molecular functional categories (a) and biological process categories (b) according to homology to genes in Uniprot with a gene ontology classification.

To predict the functions of the *C. sinensis* genes, we independently classified a total of 12,830 CsAEs into functional categories by analyzing automated gene ontology assignments [42]. The most accurate method to identify new members of known gene function among gene transcripts is to retrieve a sequence-based homology of the translated transcripts using domains extracted from a multiple alignment of gene members with known functions [43]. To functionally categorize CsAEs using domain homology searches, we translated the 12,830 CsAEs in six reading frames and recruited them into InterProScan [32], which aligned 6,106 CsAEs to InterPro entries (E-value≤1e^−4^). Among these, 2,889 CsAEs were assigned to 23,178 GO accession numbers. The 23,178 accession numbers further generated 2,349 distinguished GO mappings in two major ontologies, molecular functions and biological processes. We assigned 2,679 CsAEs (20.9%) to 17 main molecular functional subcategories and 2,195 CsAEs (17.1%) to 23 main biological process subcategories (**Figure 1**). The most abundant groups represented under the molecular function category were assigned to the GO categories of nucleotide binding (9.1%), nucleic acid binding (6.2%), ion binding (5.7%), transferase activity (6.0%), and hydrolase activity (7.4%). The most abundant groups represented under the biological process category corresponded to the GO categories of cellular component organization and biogenesis (2.3%), transport (2.9%), localization (3.0%), biosynthetic processes (2.4%), metabolic processes of nucleobases, nucleosides, nucleotides and nucleic acids (2.7%), and protein metabolism (5.2%). The apparent discrepancies between these values may be due to the fact that one InterProScan number can be assigned to more than one GO accession number, and one GO accession number can be mapped to multiple parental categories and CsAEs [33].

### Putative single nucleotide polymorphisms (SNPs)

SNPs are the most common and abundant type of genetic variation between individuals and are used in population and evolutionary biology studies [44]. In addition, SNPs can be used as markers for physical and genetic mapping. SNP discovery based on large-scale EST datasets has proven an efficient technique for identifying large numbers of SNPs. Among the 7,184 CsAE contigs, SNPs were discovered in 2,896 contigs (40%). A large majority (77%) of the SNPs were detected from contigs consisting of 2–4 ESTs, while the remainder of the SNPs were discovered in contigs consisting of more than 5 ESTs (**Table 2**). A total of 9,077 SNPs were detected from the 7,184 contigs, which is an average of 0.37 SNPs per 100 bp. The putative SNP frequency varied from 0.22 to 0.50 SNPs per 100 base pairs among contigs of different sizes. The genetic diversity of *C. sinensis* SNPs was similar to that reported for *S. japonicum*: an average of 0.35 SNPs per 100 bp [21]. In eukaryotic organisms, SNPs occur every 500 to 1,000 bp, at frequencies higher than those found in non-eukaryotic organisms [45]. Almost all of the predicted CsAEs with >0.19 SNPs/100 bp were no-match genes, and only several were homologous to *S. mansoni* genes of unknown function. In trematodes, SNPs in the first codon position can result in an amino acid substitution, which may lead to structural changes in the respective proteins and affect the formation of functional domains, antigenic epitopes, or drug binding sites [44].

**Table 2:** SNPs of *C. sinensis* identified using AutoSNP software.

No. of sequences in each contig	No. of contigs with SNPs	No. of total SNPs	Total consensus length (bp)	SNP frequency (per 100 bp)
2	950	2,398	689,883	0.35
3	760	2,396	604,004	0.40
4	520	2,267	451,608	0.50
5	128	305	112,313	0.27
6	123	300	117,116	0.26
7–10	108	212	96,884	0.22
11–20	108	279	108,014	0.26
21–30	59	171	78,977	0.22
31–50	64	226	104,073	0.22
>50	76	523	112,516	0.46
Total	2,896	9,077	2,475,388	0.37

### Abundantly expressed gene transcripts in the different developmental stages

The 30 genes most abundantly expressed were investigated further **(Table S1)**. Genes with significant *p*-values were analyzed by one-to-one comparison with the number of reads per the same gene in each stage. Eighteen genes in the adult stage and 12 genes each in the egg and metacercaria stages were annotated. Highly abundant genes should be conserved based on the reasoning that they are major constituents of the transcriptome of the organism and are therefore likely to be functional, but the highly abundant genes found in *C. sinensis* had few homologs, indicating that this organism is developmentally quite different to other organisms for which gene abundance information is available. Genes expressed abundantly across all three stages coded for ubiquitin family proteins, elongation factor 1-alpha, fructose bisphosphate aldolase, which are all proteins involved in basal and energy metabolisms. Genes highly expressed in the adult compared to the metacercaria and egg encoded structural and reproduction-associated proteins (beta-tubulin, ferritin), detoxification proteins (glutathione *S*-transferase), transportation proteins (clonorporin 1, sodium/glucose co-transporter), energy production proteins (GAPDH, mitochondrial malate dehydrogenase), and enzymes (cysteine protease, PHGPx isoform 1). In particular, cysteine proteases have previously been shown to be the most abundantly expressed proteins in *C. sinensis* adults [19], [20], [24]. In the egg stage, the gene encoding acyl-CoA synthetase long chain family member 5 (ACSL5) was highly expressed among the known genes. ACSL5 is a member of the highly conserved ACSL family. In protozoan parasites, ACSL5 is thought to catalyze the conversion of long-chain fatty acids to CoA derivatives to enable parasite growth [46]. The majority of highly expressed CsAEs was unknown or hypothetical genes and therefore deserved further study.

### Evolutionary and functionality conservation

To investigate the evolutionary and functional conservation of the transcriptome of *C. sinensis*, we estimated gene numbers and the degree of conservation among *C. sinensis* genes and the genomes of diverse eukaryotic organisms. We performed pair-wise sequence comparisons of all *C. sinensis* transcripts using BLASTX with an E-value cut-off from 1e^−10^ to 1e^−200^. The *C. sinensis* transcriptome shared 22.9% genes with *Homo sapiens*, 23.0% with *Mus musculus*, 20.5% with *Drosophila melanogaster*, 17.8% with *Caenorhabditis elegans*, and 26.6% *Schistosoma japonicum* at an E-value≤10^−20^. The CsAEs showed a moderate degree of sequence homology to the genes of the comparator organisms and a higher degree of sequence homology to *S. japonicum* genes **(** **Table 3** **).** Genes highly conserved between the CsAEs and *S. japonicum* may be important for parasite survival. These genes are discussed in more detail in the next section. A small fraction of genes (37 genes; 0.3%) were highly conserved (E-value≤10^−200^) across all the animals compared in this study **(** **Table 4** **).** These genes encode proteins such as actin, tubulin, translation elongation factor 1, valosin-containing protein, glycogen phosphorylase, and heat shock protein 70.

**Table 3:** Comparison of the transcriptomes of *C. sinensis* and selected eukaryotes.

	Unigene(%)					
Homology	*Homo sapiens*	*Mus musculus*	*Drosophila melanogaster*	*Caenorhabditis elegans*	*Schistosoma japonicum*	All organisms+
**E-value≤1e^−200^**	19(0.2%)	18(0.2%)	16(0.2%)	18(0.2%)	16(0.2%)	37(0.3%)
**E-value ≤1e^−100^**	158(1.6%)	159(1.6%)	137(1.4%)	114(1.3%)	205(2.0%)	346(3.0%)
**E-value ≤1e^−50^**	905(8.6%)	906(8.7%)	733(7.6%)	564(6.2%)	1,125(11.2%)	1,674(14.7%)
**E-value ≤1e^−30^**	1,696(16.5%)	1,687(16.4%)	1,325(14.4%)	1,078(12.3%)	1,936(20.4%)	2,850(26.0%)
**E-value ≤1e^−20^**	2,305(22.9%)	2,302(23.0%)	1,821(20.5%)	1,468(17.8%)	2,424(26.6%)	3,654(33.9%)
**E-value ≤1e^−10^**	3,110(31.7%)	3,092(31.8%)	2,396(28.2%)	1,946(24.9%)	2,910(33.3%)	4,643(43.6%)
**Little homology** *	3,477(35.4%)	3,412(35.2%)	2,669(31.4%)	2,193(28.2%)	3,028(35.0%)	5,269(49.1%)

**+:** EST data were searched against the entire protein database by BLASTX.

*More than 25% homology with at least 30 amino acid residues deduced from CsAEs.

%the percent ratio of matched clusters of total 12,830 CsAEs to the protein database of model eukaryotes.

**Table 4:** Ultraconserved contigs of *C. sinensis.*

Accession No.	Descriptions	*H. sapiens*	*M. musculus*	*D. melanogaster*	*C. elegans*	*S. japonicum*	All
CAO79607.1	Beta-tubulin	○	○	○	○	○	○
AAQ16109.1	Elongation factor1-alpha	○	○	○	○	○	○
ABS52704.1	Heat shock protein 70	○	○	○	○	-	○
NP_001086877.1	Translation elongation factor 2	○	○	○	○	-	○
ABS81352.1	Phospho glucose isomerase	○	○	○	○	-	○
AAH83344.1	Tubulin, alpha1A	○	○	○	○	○	○
CAQ13492.1	Propionyl CoenzymeA carboxylase, beta polypeptide	○	○	-	○	-	○
AAW27581.1	SJCHGC09453 protein	○	○	○	○	○	○
AAI61792.1	Unknown	○	○	○	○	-	○
AAS93901.1	Glycogen phosphorylase	○	○	○	○	○	○
AAM69406.1	Heat shock protein HSP60	○	○	○	○	○	○
BAB84579.1	Actin2	○	○	○	○	○	○
AAW27659.1	SJCHGC00820 protein	○	○	○	○	○	○
XP_001118968.1	Glucan(1,4-alpha-), branching enzyme1	○	○	○	○	-	○
XP_001107041.1	Oxoglutarate dehydrogenase-like isoform2	○	○	○	○	-	○
XP_001089681.1	ATP synthase, H+ transporting, mitochondrial F1 complex, alpha subunit	○	○	○	○	-	○
XP_001627515.1	Predicted protein	-	○	○	-	-	○
AAW27320.1	SJCHGC06322 protein	-	-	-	-	○	○
AAW27782.1	SJCHGC00653 protein	-	-	-	-	○	○
AAW26140.1	SJCHGC02536 protein	-	-	-	-	○	○
AAX27366.2	SJCHGC05847 protein	-	-	-	-	○	○
AAW27345.1	SJCHGC09272 protein	-	-	-	-	○	○
AAW26056.1	SJCHGC05577 protein	-	-	-	-	○	-
AAW27129.1	SJCHGC06305 protein	-	-	-	-	○	-
XP_001177707.1	Gag-polpoly protein	-	-	-	-	-	○
XP_001628202.1	Predicted protein	-	-	-	-	-	○
XP_001952362.1	Zinc finger protein	-	-	-	-	-	○
ABI26619.1	Enolase	-	-	-	-	-	○

*Cut-off value <1e^−200^.

### *Clonorchis sinensis* and parasitism

**Figure 2:**
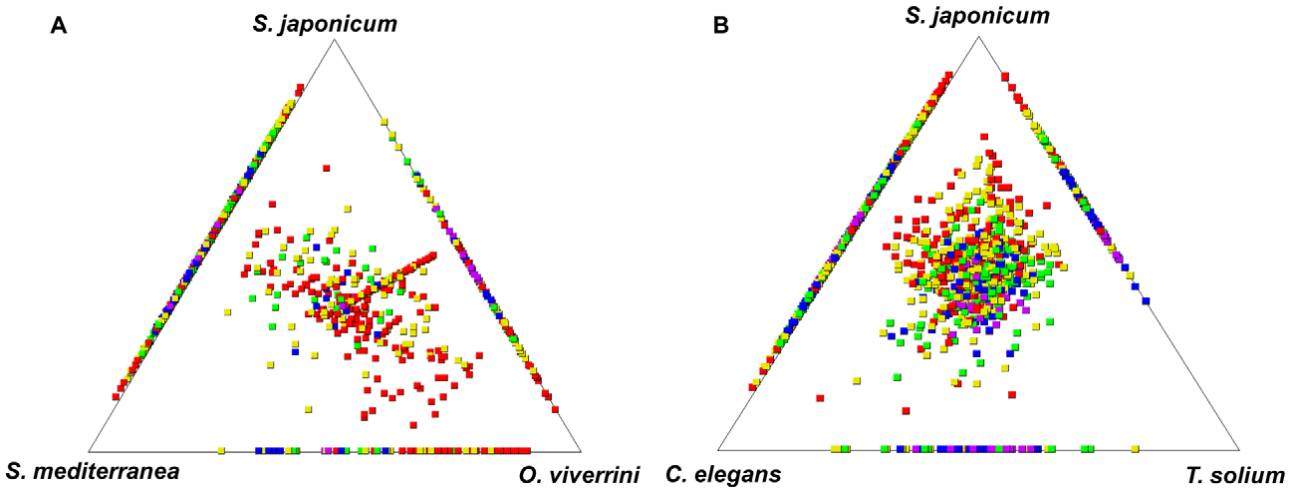
Global relative similarity between *C. sinensis* and other species analyzed at the whole transcriptome scale . Each *C. sinensis* contigs and singlets were searched against the whole transcriptome using TBLASTX score (a cut-off of ≥50). Similarity comparison of parasitic organisms with a free-living flatworm (A) or with free-living nematode (B). Square tiles indicate genes, with the squares colored by their highest TBLASTX score to each of the databases: red ≥300; yellow ≥200; green ≥150, blue ≥100 and purple <100.

SimiTri graphically displays the relative similarity of one organism to others using bulk datasets from the respective organisms. The degree of similarity among helminth sequences was determined using two-dimensional plots [40]. We used SimiTri to analyze the global relative similarity of CsAEs to other parasitic or free-living trematodes, cestode and nematode (**Figure** **2**). *C. sinensis* (12,830 CsAEs) was more similar to the two parasitic flukes, *S. japonicum* and *O. viverrini*, than to the free-living helminths *Schmidtea mediterranea* (73,650 ESTs) and *C. elegans* (474,350 ESTs). When compared to both *Opisthorchis viverrini* (4,194 ESTs) and *S. japonicum* (99,069 ESTs), the closest neighbor of *C. sinensis* was *O. viverrini*, consistent with the general taxonomic classification of these trematodes and the recent molecular phylogeny of Digenean trematodes based on morphological characters and the sequence of the nuclear ribosomal small subunit (18S) [47].

Based on the SimiTri analyses results, 23 CsAEs were considered to contain parasitism-related genes of *C. sinensis* (**Table S2)**. The genes encoded by these 23 CsAEs showed higher sequence similarity to the parasitic platyhelminths, *O. viverrini*, *S. japonicum*, and *Taenia solium* than to free-living ones, *S. mediterranea* and *C. elegans* (Figures 2A and B). Of these, 12 CsAEs had an unknown function, whereas 11 CsAEs had cell communication, ion transport, metabolic processes, nucleotide and protein binding, or oxidoreduction functional annotations.

### Membrane proteins, channels and transporters

#### Channels

In the adult *C. sinensis* cDNA library, EST encoding K^+^-channel was abundant but that of Ca^+2^-channel was rare **(** **Table 5**). No Na^+^ -channel EST was found in any of the three developmental stages. From an evolutionary point of view, K^+^-channels are ancient, occurring in all three domains of life, and are abundant in invertebrate animals. K^+^-channels are ubiquitous and maintain cell homeostasis in organisms. Ca^+2^-channels emerged later in evolutionary time. In more basal animals, Ca^+2^-channels are activated in response to action potentials of nerve systems and provoke slow actions and movements. Na^+^-channels are more elaborate and are often multimeric, and are generally rare in invertebrates compared to higher vertebrate animals [48]. The frequency distribution of these cation channels is consistent with phylogenetic placement of *C. sinensis* in the tree of life. Specific structural motifs in the interacting domains of the beta subunit of Ca^+2^ channels render flukes PZQ susceptible. These specific structural motifs were found in the beta-subunits of *C. sinensis* adults, which explain why they are vulnerable to PZQ.

**Table 5:** Developmental expression of ion-channels and transporters in *C. sinensis.*

		No. of reads		
Category	Description	Adult	Metacercaria	Egg
**K^+^-channel**	Potassium channel protein	10	4	2
**Ca^+2^-channel**	High voltage-gated Ca^+2^-channel, batasubunit,CavB	2	2	1
	High voltage-activated Ca^+2^-channel, CavA	0	1	0
**Na^+^-channel**	Amiloide-sensitive sodium channel-related protein	1	0	0
**Cl^−^-channel**	Chloride channel protein 7	7	4	1
**Sodium Transporter**	Solute carrier family 17 (sodium phosphate)	2	0	0
	Solute carrier family 5 (sodium/glucose cotransporter)	77	0	0
	Sodium/sialic acid cotransporter, putative	2	0	0
	Sodium/bile acid cotransporter	3	14	0
	Potassium-dependent sodium-calcium exchanger	1	0	0
	Sodium/hydrogen exchanger 7, 9	1	0	0
	Sodium/myo-inositol cotransporter	1	0	0
	Sodium/proton exchanger 3	1	0	0
**Na^+^/K^+^-ATPase**	Na^+^/K^+^-transporting ATPase beta	4	0	0
	Na^+^/K^+^-transporting ATPase beta-2 chain	1	0	0
**Glucose transporter**	Glucose transporter	47	0	20
	Sodium/glucose co-transporter	77	0	0
**Amino acid transporters**	Amino acid transporter	22	2	9
**Zinc transporter**	Zinc transporter	15	5	4
**Phosphate transporter**	Glycerol-3-phosphate transporter	2	2	3
	Phosphate transporter	2	0	1
**Fatty acid transporter**	Fatty acid transporter, member 1	16	18	2

#### Transporters

A large number of glucose transporters were found in both the adult and egg stages **(** **Table 5** **).** Glucose/sodium co-transporters and Na^+^/K^+^-transporting ATPases were abundant only in adult *C. sinensis*, not in the metacercaria. This fluke species consumes large amounts of glucose to generate energy and metabolic intermediates for physiologic regulation. In adult *C. sinensis*, glucose appears to be imported actively from the environment through glucose/sodium co-transporters using ATP as an energy source. Glucose molecules may move passively through the glucose transporters between cells in fluke tissues. Anion channel proteins including chloride channels were 4-fold more frequent than cationic channels, which could compensate for the large amount of Na^+^ ions co-imported with exogenous glucose. These anionic channels may be suitable targets for vaccine or drug development.

CsAEs coding for bile acid beta-glucosidase and a sodium-bile acid cotransporter, a component of the bile acid transportation pathway, were present in the *C. sinensis* EST pool **(** **Table 5** **)**. Bile acid beta-glucosidase converts bile acid to a soluble conjugated form to facilitate its secretion. The sodium-bile acid cotransporter, which imports bile salts with Na^+^-dependency, was abundantly expressed in the metacercaria stage. This type of transporter is responsible for the influx of conjugated bile acids into hepatocytes, ileal enterocytes, and cholangiocytes in mammals [49], [50]. The presence of these transporters in *C. sinensis* suggests that *C. sinensis* thrives in bile juice by utilizing bile acid and its derivatives for normal physiologic pathways. *C. sinensis* is expected to have a bile acid exporting system comprising bile salt export pumps, organic solute transporters, and/or multidrug resistance protein 2 to maintain cellular homeostasis [49], [50].

#### Neurotransmitters & receptors

CsAEs encoding proteins involved in neurotransmission such as serotonin receptors, tryptophan hydroxylase, aromatic amino acid decarboxylase, glutamate receptors, glutaminase, GABA receptor-associated proteins, acetylcholine esterase, and DOPA-decarboxylase were found as rare species **(Table S3)**. The presence of these neurotransmission-related proteins implies that *C. sinensis* has a web of serotoninergic, glutaminergic, and cholinergic neurons, which is consistent with the previous observation that the nervous systems of trematodes are highly conserved [51].

### Proteases and protease inhibitors

**Figure 3:**
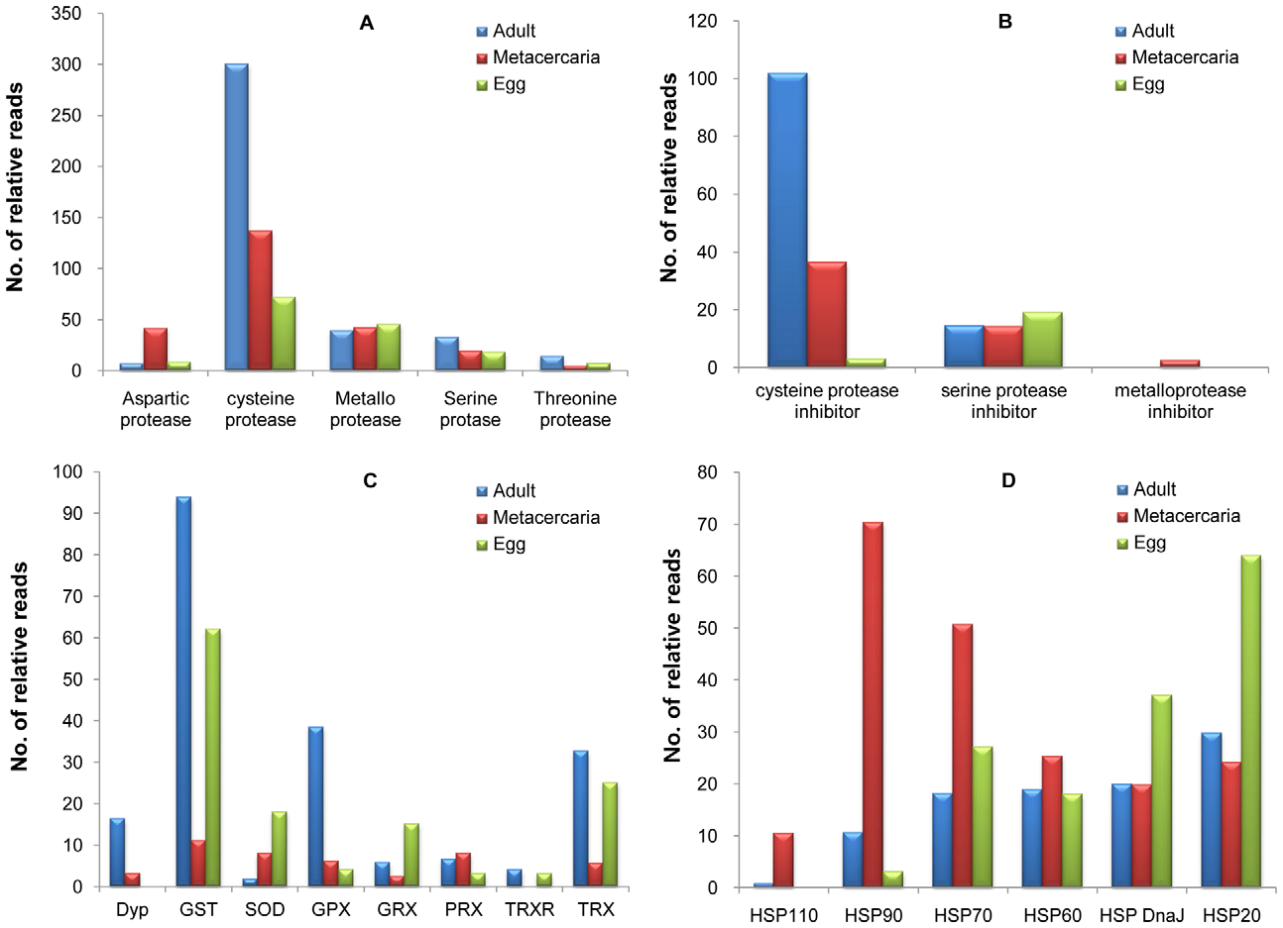
Developmental expression of proteases and protease inhibitors, antioxidant enzymes, and heat shock proteins in *C. sinensis*. Relative reads refer to the number of calculated reads in proportion to the read size for each developmental stage (adult: metacercaria : egg = 2.76∶1.62∶1). (A) Proteases, (B) protease inhibitors, (C) antioxidant enzymes, (D) stress response proteins. Dyp, dye-decolorizing peroxidase; GST, glutathione-s-transferase; SOD, superoxide dismutase; GPX, glutathione peroxidase; GRX, glutaredoxin; PRX, Peroxiredoxin; TRXR, thioredoxin reductase; TRX, thioredoxin; HSP, heat hock protein.

Proteases of parasitic origin are known to be important virulence factors based on genomic and proteomic analysis of several major global helminth species [52], [53], [54]. Secretory proteases of parasites are ubiquitous enzymes that have been implicated in several diverse physiological and adaptive mechanisms, such as tissue penetration, larval migration, immunoevasion, digestion, and excystation [55]. Because these proteases are indispensable for parasite viability and growth, they have been suggested as potential targets for vaccines or chemotherapeutic agents [56], [57]. We classified the CsAE proteases into four functional groups based on the catalytic type, namely serine, threonine, aspartate, and metallo- or cysteine proteases (Figure 3A). Cysteine proteases showed the highest expression (68.8%) levels among the four types of proteases during all three developmental stages. Cysteine proteases of *C. sinensis* are developmentally controlled and essential for survival because they are involved in processes such as nutrient uptake, tissue invasion, and evasion from host immune attacks [58]. Of the *C. sinensis* cysteine proteases, the cathepsin F-like isoenzyme (CsCF-6) was expressed across all developmental stages, and we observed that transcript levels of this protein increased according to the developmental stage of the parasite [59]. Metalloproteases were detected in all three developmental stages of *C. sinensis* **(** **Figure 3A** **).** Metalloproteases are crucial proteases for invasion and immune evasion of flukes in addition to the general roles they play in catabolic reactions and protein processing [57].

Parasites utilize protease inhibitors to survive in their hosts; protease inhibitors can prevent damage by mature proteases prior to their secretion from the parasite and protect them against the digestive proteases of the hosts [60]. Diverse proteins function as protease inhibitors, but they have a common biochemical mechanism and are characterized by rapid evolution of their sequences [61]. In our study, cysteine protease inhibitor expression was highest in the adult stage among all three stages (Figure 3B). Cysteine protease inhibitors (cystatins) regulate cysteine proteases and modulate host immune responses [60]. Cystatins of *C. elegans* have been shown to inhibit cathepsin B while filarial cystatins have been shown to inhibit the proliferation of murine and human T-cells. Because *C. sinensis* cysteine proteases are expressed most abundantly in the intestinal epithelium for uptake of nutrients [59], cystatins could be expressed in the intestinal epithelium to fine-tune the intracellular activities of cysteine proteases. Expression of serine protease inhibitors remained stable in all stages with slightly greater expression levels observed in the egg stage **(** **Figure 3B** **)**. This finding is consistent with a previous study that demonstrated that serine protease inhibitors were present mainly in the eggs of *C. sinensis* [62]. Transcripts encoding metalloprotease inhibitors were found only in the metacercaria library and inhibitors of aspartic and threonine proteases were not identified in any of the developmental stages.

### Antioxidant enzymes

Several antioxidant enzymes constituting the oxidoreduction system were encoded by the CsAEs, with the expression levels of the various enzymes varying according to developmental stage **(** **Figure 3C** **)**. Antioxidant enzymes catalyze reactions that neutralize endogenous and exogenous reactive oxygen species (ROS) that are produced either by aerobic cellular metabolism or by host immune responses [63]. Regulation of the expression levels of these enzymes during each development stage is therefore important to cope with host-produced ROS [64]. In the *C. sinensis* transcriptome, glutathione-*S*-transferases (GSTs) were the most highly expressed antioxidant enzymes, especially in the adult and egg stages. In *Fasciola hepatica*, GSTs are expressed at much lower levels in juvenile worms than in adult worms living in the bile duct, implying that adult worms require more protection against host immune responses [63]. In *C.* s*inensis,* two GST isoenzymes have been identified: a 26 kDa GST and a 28 kDa GST [65]. Glutathione peroxidase (GPx) and thioredoxins (TRX) were expressed differentially at high levels in the adult stage (Figure 3C). *C. sinensis* GPx has been reported to be specifically localized in the vitellocytes of vitelline glands and in the premature eggs [66]. GPx defends against ROS and repairs ROS-induced damage in trematodes that do not have catalases [53]. TRX was highly expressed (Figure 3). In *Schistosoma mansoni*, TRX is secreted from eggs and plays a crucial role in protecting eggs from host-induced ROS production [63]. All antioxidant enzymes were expressed at low levels in the metacercaria stage (Figure 3), which can be explained by the fact that this is a dormant state with a depressed metabolism that is protected by a cyst wall from exogenous oxidative stresses.

### Stress responsive genes

Heat shock proteins (HSPs) function as molecular chaperones and play an important role in the stress response to a variety of biological stresses such as heat shock, hypoxia, mechanical stimuli, lack of glucose, and UV exposure by assisting in the refolding of denatured proteins into active forms or targeting them for degradation [67]. We identified six HSPs from the CsAEs: HSP110, HSP90, HSP70, HSP60, HSP DnaJ, and HSP20. Most HSPs were expressed at higher levels in the metacercaria and egg stages than in adult worms. The higher molecular weight HSPs such as HSP110, HSP90, HSP70 and HSP60 were more highly expressed in the metacercaria stage, while the lower molecular weight HSPs, HSP DnaJ and HSP20, were expressed at higher levels in the egg stage. In *C. sinensis* adults, HSPs were expressed at low levels relative to the metacercaria and egg stages (**Figure 3D**). In the life cycle of *C. sinensis,* the metacercariae experience a large thermal change when they move from the environment (ambient temperature) to the stomach of the mammalian host (37°C). Furthermore, as the metacercariae pass down and excyst in the duodenum, they face the osmotic stress of intestinal secretions, and then when they migrate into the bile duct, they are exposed to bile juices. The higher molecular weight *C. sinensis* HSPs are likely to responsd to these thermally- and environmentally-induced stresses [68], [69]. The *C. sinensis* eggs that ovulate in the bile duct are carried down the intestine and passed out in the feces of the mammalian host into the environment, thereby experiencing cold shock. In the *C. sinensis* eggs, the low molecular weight HSPs may function to protect the eggs against cold thermal shock [70], while the high molecular weight HSPs might contribute to recovery from the cold shock [71].

### Cell proliferation and cholangiocarcinoma-related genes

The *C. sinensis* transcriptome had several contigs encoding proteins associated with cell proliferation and apoptosis such as granulin, epidermal growth factor (EGF), tumor growth factor (TGF) interacting protein, and inhibitors and regulators of apoptosis. Among these proteins, granulin, encoded for by 67 reads, was most abundantly expressed with a 8.6-fold higher expression level in adults than metacercariae. Similarly, the EGF gene was also expressed at 2.5-fold higher levels in the adult stage than the metacercaria stage (Table 6). Transcriptomic datasets of *C. sinensis* and *O. viverrini* were previously analyzed for proteins common to carcinogenesis and a large number of the amino acid sequences of these trematodes were inferred to have homologs to genes involved in human cancer development [25]. The *C. sinensis* genes associated with apoptosis, cell proliferation, and cancer development encoded laminins, c-Jun N-terminal kinase, catenins, cyclin-dependent kinases, histone deacetylases, MFS transporters, serine/threonine kinases, and transcription factors **(** **Table 6** **).** *C. sinensis* infection provokes both acute and chronic pathological changes such as proliferation of the bile duct epithelium and periductal fibrosis that is disseminated over the biliary tree from proximal to remote biliary capillaries [5]. The mitogen-like proteins secreted or excreted from *C. sinensis* are likely to be provocative agents that cause biliary epithelial alterations, as has been documented for the granulin-like growth factor of *O. viverrini* [72], [73]. Proliferating cholangiocytes could be vulnerable to DNA damage from endogenous and exogenous carcinogens, bioreactive free radicals, and nitrosocompounds. The apoptosis inhibitor and regulatory proteins identified in the transcriptome of *C. sinensis* in this study (Table 6) may prevent the death of DNA-damaged cells and possibly facilitate their transformation into cancerous cells [73]. Epidemiologically, the incidence of cholangiocarcinoma is significantly higher in clonorchiasis endemic areas than in non-endemic areas [74]. Experimentally, infection with *C. sinensis* and the ingestion of dimethylnitrosamine by Syrian golden hamsters resulted in cholangiocarcinoma [9], [75].

**Table 6:** Expression of genes associated with apoptosis, cell proliferation, and cancer development in *C. sinensis.*

Category	Description	No. of reads		
		Adult	Metacercaria	Egg
**Apoptosis**	Inhibitor of apoptosis protein	9	8	0
	Apoptosis-linked gene 2 protein	1	3	9
	Cell-cycle and apoptosis regulatory protein 1	14	8	0
**Cell proliferation**	Granulin	7	4	2
	Granulin precursor (Proepithelin) (PEPI)	36	1	17
	EGF	1	2	0
	EGF/Laminin	9	2	13
	Multiple EGF-like-domains 6	0	2	0
	TGF-beta receptor interacting protein 1	2	3	0
**Cancer development** *	c-Jun N-terminal kinase	23	11	0
	Catenin (cadherin-associated protein)	5	15	8
	Axin 1	0	3	0
	Cell division control protein 42	2	2	3
	Cyclin-dependent kinase	11	4	3
	Death-associated protein kinase	2	0	0
	DNA mismatch repair protein	3	2	0
	DNA repair protein	10	0	0
	Growth factor receptor-binding protein	1	1	4
	Histone deacetylase	12	9	9
	Integrin beta	12	3	0
	Laminin	8	4	13
	MFS transporter, SP family, solute carrier family	77	0	0
	Mitogen-activated protein kinase kinase	6	0	2
	RING-box protein 1	9	0	0
	Serine/threonine-protein kinase	32	29	23
	Transcription elongation factor	8	1	2
	Transcription factor	27	20	15
	Tropomyosin	30	38	12

*Refer to Reference No. 25.

### Drug target candidates

Praziquantel is currently used to treat clonorchiasis. However, the efficacy of praziquantel against clonorchiasis has been reported to be poor in northern Vietnam [76]. Tribendimidine has recently emerged as a promising alternative to praziquantel for the treatment of human opisthorchiasis [77]. Transcriptomic datasets facilitate the search for new drug targets by mining them with bioinformatic tools that employ statistical and network analyses, parasite-specific physico-metabolic pathways and developmental regulation can be found. Membrane proteins including channels, transporters, and permeases, which play important roles in host-parasite interactions, were some of the first novel targets identified using transcriptome data [78], [79]. A total of 435 CsAEs that had more than two transmembrane domains were screened using the TMHMM algorithm [36], and the localization of the predicted proteins was determined using PSORTb based on homology to proteins of known localization [35]. CsAEs homologous to proteins of the free-living flatworm *S. mediterranea*, or of humans were selected using a cut-off E-value≤1e^−10^ using BLASTX. Five genes not present in vertebrates were identified as putative drug targets using the screening strategy described above (Table S4). Tetraspanins, four-transmembrane-domain proteins, are present on the outer tegument of trematodes and function as receptors for host molecules [80]. Tetraspanins are a recognized vaccine target for *S. mansoni* [78]. ADP ribosylation-like factor-6 (ARL6) interacting protein, which interacts with the ARL6 protein [81], is involved in hematopoietic maturation processes such as protein transport, membrane trafficking, and cell signaling [82]. Myelin proteolipid protein is a transmembrane protein that has been suggested to serve as a structural component of myelin, contributing to both the stability and compact lamellar structure of myelin [83].

### Diagnostic antigen candidates

Serodiagnostic methods are used to screen for patients infected with *C. sinensis* and as supportive diagnosis tools in individual patients. Antigen proteins have been identified from the excretory-secretory products of *C. sinensis* and have been purified from crude extracts [84]. As antigenic preparations, crude extracts of *C. sinensis* show high sensitivity but low specificity toward the sera of clonorchiasis patients. In contrast, some of the recombinant antigenic proteins used for screening have high specificity but low sensitivity [84]. Potent serodiagnostic antigens for clonorchiasis are therefore still lacking. As the first step to find antigen candidates, the *C. sinensis* transcriptome data was filtered for secretory signal peptides using the SignalP 3.0 server and a neural network/hidden Markov model [34], while proteins secreted into the extracellular space were searched for using PSORTb [35]. After removing proteins with transmembrane domains using TMHMM, 411 CsAEs were designated ‘secretory’. To determine which of these proteins is potentially antigenic [36], the secretory candidates were filtered using the ABCpred server [85] with default parameters, and 43 CsAEs with two or more B-cell epitopes and a score of 0.82 or higher were obtained. By removing proteins homologous to mammalian and nuclear proteins, a total of 19 CsAEs were identified as putative antigen candidates. Examples of these candidates include a male sterility protein, cathepsin L-like cysteine proteinase A, protein disulfide isomerase-related protein P5 precursor, cryptosporidial mucin, and TGF-beta receptor interacting protein 1 **(** **Table 7** **)**. Recombinant antigenic proteins encoded by the selected CsAEs could be synthesized *in vitro* using a high-throughput cell-free system [86], and their antigenicity for the serodiagnosis of clonorchiasis could be determined using various methods such as a protein chip [87].

**Table 7:** Putative serodiagnostic antigens.

EST ID	Description
CL1051Contig1	Male sterility protein
CSA22042	Cathepsin L-like cysteine proteinase A
CL1502Contig1	Protein disulfide isomerase-related protein P5 precursor
CL2119Contig1	LAG1 (longevity assurance homolog)
CL2671Contig1	Flagelliform silk protein
CL11Contig2	Cryptopsoridial mucin, large thr stretch, signal peptide sequence
CSM15703	TGF-beta receptor interacting protein 1
CSA19952	DJ-1_PfpI
CL3175Contig1	Eukaryotic translation initiation factor 4A2 isoform 1
CL1573Contig1	Predicted protein
CL3180Contig1	Peptidyl-prolyl cis-trans isomerase B precursor (PPIase B) (Cyclophilin B)
CL6354Contig1	UDP-GlcNAc:betaGal beta-1,3-N-acetylglucosaminyltransferase 7
CL6614Contig1	Oxysterol binding protein
CL5658Contig1	Vesicular mannose-binding lectin
CL222Contig1	Glycoprotein X precursor
CSA21481	Stromal interaction molecule 2
CSA01410	Calreticulin precursor (SM4 protein)
CL31Contig2	Melibiase family protein
CSA17420	Group 14 allergen protein

## Supporting Information

Figure S1 Contigs and singlets of the assembled *C. sinensis* EST pool according to developmental stage.
(TIFF)

Figure S2 **The annotation of CsAEs using Interpro and BLASTX.** Whole 12,830 CsAEs were searched for homologs in the NCBI NR database and the InterPro database, and the retrieved homologs were manually curated. A total of 7,132 CsAEs were annotated with homologs, but the remaining 5,698 (orange) found to have no homolog. Of the annotateds, 5,387 CsAEs (violet) matched homologs in both databases, 719 ones (green) in the InterPro, and 1,026 ones (blue) in the BLASTX.
(TIFF)

Table S1 The 30 most abundantly expressed genes in the adult, metacercaria, and egg stages of *C. sinensis*.
(DOC)

Table S2 Putative parasitism-related genes of *C. sinensis*.
(DOC)

Table S3 CsAEs of neuro-receptors and neurotransmitter producing enzymes according to *C. sinensis* developmental stage.
(DOC)

Table S4 Putative drug targets of *C. sinensis*
(DOC)
