# Supplementary figures and images for: Developmental Transcriptomic Features of the Carcinogenic Liver Fluke, Clonorchis sinensis
Source: PLoS Negl Trop Dis. 2011 Jun 28;5(6):e1208. doi: 10.1371/journal.pntd.0001208 (PMC3125140; doi:10.1371/journal.pntd.0001208)

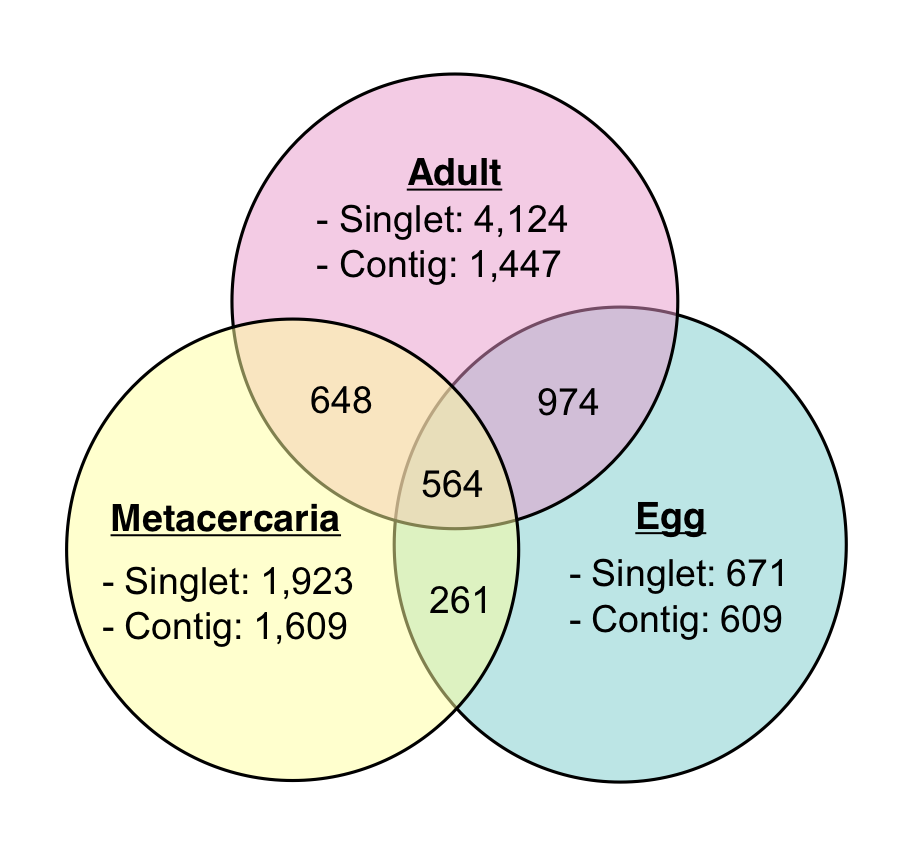

Supplement: Figure S1 — Contigs and singlets of the assembled C. sinensis EST pool according to developmental stage. (TIFF) [file pntd.0001208.s001.tiff]

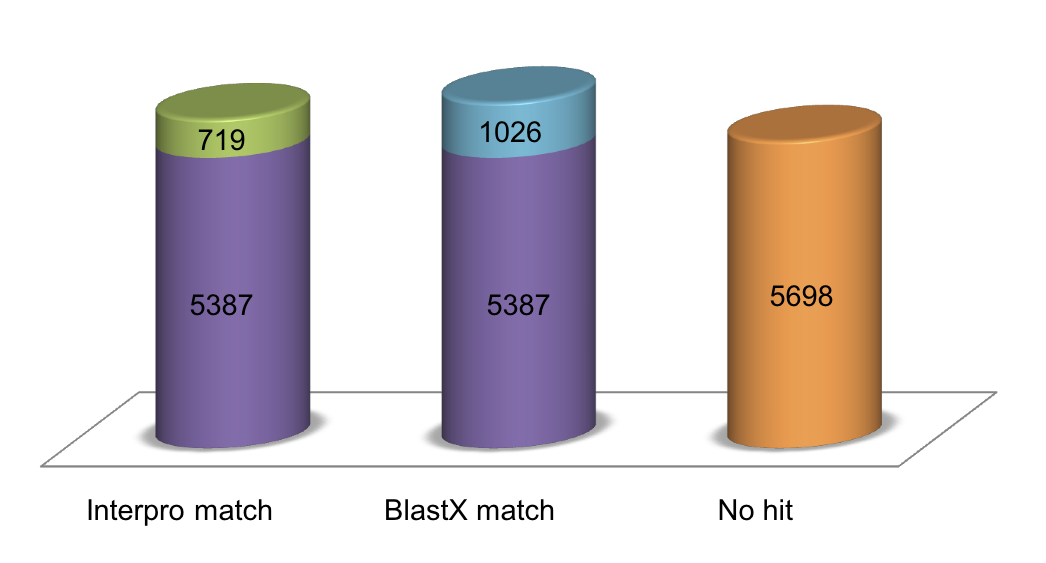

Supplement: Figure S2 — The annotation of CsAEs using Interpro and BLASTX. Whole 12,830 CsAEs were searched for homologs in the NCBI NR database and the InterPro database, and the retrieved homologs were manually curated. A total of 7,132 CsAEs were annotated with homologs, but the remaining 5,698 (orange) found to have no homolog. Of the annotateds, 5,387 CsAEs (violet) matched homologs in both databases, 719 ones (green) in the InterPro, and 1,026 ones (blue) in the BLASTX. (TIFF) [file pntd.0001208.s002.tiff]
