# Supplementary material for: Developmental Transcriptomic Features of the Carcinogenic Liver Fluke, Clonorchis sinensis
Source: PLoS Negl Trop Dis. 2011 Jun 28;5(6):e1208. doi: 10.1371/journal.pntd.0001208 (PMC3125140; doi:10.1371/journal.pntd.0001208)
Supplement: Table S1 — The 30 most abundantly expressed genes in the adult, metacercaria, and egg stages of C. sinensis. (DOC) [file pntd.0001208.s003.doc]

**Table S1. The 30 most abundantly expressed genes in the adult, metacercaria, and egg stages of *C. sinensis***

| **Rank** | **Adult** | | | 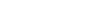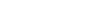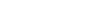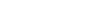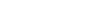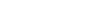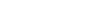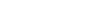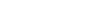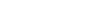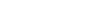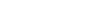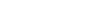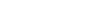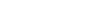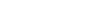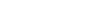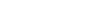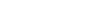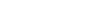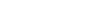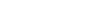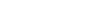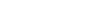**Metacercaria** | | | **Egg** | | |
| --- | --- | --- | --- | --- | --- | --- | --- | --- | --- |
|  | **Gene description** | **Organism** | **Reads (%)** | **Gene description** | **Organism** | **Reads (%)** | **Gene description** | **Organism** | **Reads (%)** |
| 1 | Cysteine protease | *Clonorchis sinensis* | 329(1.22)^*+^ | unknown | unknown | 544(3.43)^+‡^ | unknown | unknown | 174(1.77)^*‡^ |
| 2 | Unknown | unknown | 305(1.13)^*+^ | unknown | unknown | 335(2.11)^+‡^ | acyl-CoA synthetase long-chain family member 5, isoform CRA_e | *Homo sapiens* | 94(0.96)^*‡^ |
| 3 | Tubulin, alpha 1A | *Mus musculus* | 280(1.04)^*+^ | SJCHGC02792 protein | *Schistosoma japonicum* | 222(1.4)^+‡^ | SJCHGC06322 protein | *Schistosoma japonicum* | 83(0.85)^*^ |
| 4 | Ubiquitin family member (ubq-1) | *Caenorhabditis elegans* | 216(0.8) | myosin heavy chain | *Schistosoma mansoni* | 126(0.79) | ENSANGP00000023128 | *Nasonia vitripennis* | 82(0.84)^*‡^ |
| 5 | Myoglobin | *Clonorchis sinensis* | 177(0.65)^*+^ | SJCHGC00820 protein | *Schistosoma japonicum* | 108(0.68)^+‡^ | elongation factor-1 | *Clonorchis sinensis* | 61(0.62) |
| 6 | LIMPETin | *Schistosoma mansoni* | 173(0.64)^*+^ | unknown | unknown | 93(0.59)^+‡^ | cytoplasmic antigen 1 | *Spirometra erinaceieuropaei* | 60(0.61)^*^ |
| 7 | beta-Tubulin | *Fasciola hepatica* | 171(0.63)^*+^ | unknown | unknown | 81(0.51)^+‡^ | SJCHGC07049 protein | *Schistosoma japonicum* | 57(0.58)^*‡^ |
| 8 | Unknown | unknown | 125(0.46)^*+^ | SJCHGC01894 protein | *Schistosoma japonicum* | 70(0.44)^+‡^ | predicted protein | *Nematostella vectensis* | 54(0.55)^*^ |
| 9 | SJCHGC09647 protein | *Schistosoma japonicum* | 122(0.45)^*+^ | SJCHGC06322 protein | *Schistosoma japonicum* | 65(0.41)^+^ | SJCHGC06778 protein | *Schistosoma japonicum* | 52(0.53)^*‡^ |
| 10 | Receptor for activated PKC | *Schistosoma mansoni* | 118(0.44)^*+^ | GAPDH | unknown | 58(0.37)^‡^ | AF272975 smoothelin-C | *Schistosoma japonicum* | 52(0.53)^*^ |
| 11 | Colloid protein | *Ornithorhynchus anatinus* | 99(0.37)^*+^ | unknown | unknown | 57(0.36)^+‡^ | unknown | unknown | 52(0.53)^*‡^ |
| 12 | SJCHGC01769 protein | *Schistosoma japonicum* | 94(0.35)^*+^ | ubiquitin | *Geodia cydonium* | 50(0.32) | ribosomal protein S3 | *Schistosoma japonicum* | 51(0.52)^‡^ |
| 13 | SJCHGC06288 protein | *Schistosoma japonicum* | 92(0.34)^*+^ | ribosomal protein L4-like protein | *Schistosoma mansoni* | 49(0.31)^+‡^ | fructose bisphosphate aldolase | *Schistosoma bovis* | 48(0.49) |
| 14 | Clonorporin 1 | *Clonorchis sinensis* | 91(0.34)^*+^ | SJCHGC06312 protein | *Schistosoma japonicum* | 49(0.31)^+‡^ | SJCHGC09192 protein | *Schistosoma japonicum* | 43(0.44)^*‡^ |
| 15 | Glutathione transferase | *Clonorchis sinensis* | 90(0.33)^*+^ | fructose bisphosphate aldolase | *Schistosoma bovis* | 46(0.29) | SJCHGC02833 protein | *Schistosoma japonicum* | 40(0.41) |
| 16 | Unknown | unknown | 87(0.32)^*+^ | unknown | *Clonorchis sinensis* | 43(0.27)^+‡^ | ubiquitin-conjugating enzyme E2Q | *Homo sapiens* | 37(0.38)^*‡^ |
| 17 | Ferritin | *Clonorchis sinensis* | 86(0.32)^*+^ | elongation factor-1 | *Clonorchis sinensis* | 42(0.26) | Y-box binding protein | *Schistosoma mansoni* | 36(0.37)^*^ |
| 18 | GAPDH | unknown | 84(0.31)^*^ | 14-3-3 epsilon | *Schistosoma mansoni* | 42(0.26)^+‡^ | Hexokinase | unknown | 33(0.34) |
| 19 | Elongation factor-1 | *Clonorchis sinensis* | 80(0.3) | unknown | unknown | 38(0.24)^+‡^ | unknown | unknown | 32(0.33)^*‡^ |
| 20 | Sodium/glucose cotransporter | *Crassostrea gigas* | 77(0.28)^*+^ | mitochondrial malate dehydrogenase | *Clonorchis sinensis* | 37(0.23)^‡^ | SJCHGC02221 protein | *Schistosoma japonicum* | 31(0.32) |
| 21 | Fructose bisphosphate aldolase | *Schistosoma bovis* | 77(0.28) | Putative high mobility group B2 protein | *Schistosoma mansoni* | 37(0.23)^+‡^ | unknown | unknown | 30(0.31) |
| 22 | SJCHGC05684 protein | *Schistosoma japonicum* | 73(0.27)^*+^ | cytoplasmic antigen 1 | *Spirometra erinaceieuropaei* | 36(0.23)^+^ | SJCHGC06078 protein | *Schistosoma japonicum* | 30(0.31) |
| 23 | SJCHGC02792 protein | *Schistosoma japonicum* | 72(0.27)^*+^ | unknown | unknown | 36(0.23)^+‡^ | LOC571499 protein | *Danio rerio* | 30(0.31)^*‡^ |
| 24 | SJCHGC06322 protein | *Schistosoma japonicum* | 70(0.26)^*+^ | 60S ribosomal protein L34-like protein | *Schistosoma mansoni* | 34(0.21)^+‡^ | ribosomal protein L4 | *Taenia asiatica* | 29(0.3)^*^ |
| 25 | PHGPx isoform 1 | *Clonorchis sinensis* | 67(0.25)^*+^ | SJCHGC03433 protein | *Schistosoma japonicum* | 34(0.21)^+‡^ | AUT1 | *Schistosoma mansoni* | 29(0.3)^*‡^ |
| 26 | Unknown | unknown | 66(0.24)^*+^ | AB063189 annexin B13a in Bombyx mori | *Schistosoma japonicum* | 32(0.2)^+‡^ | SJCHGC09416 protein | *Schistosoma japonicum* | 28(0.29) |
| 27 | Mitochondrial malate dehydrogenase | *Clonorchis sinensis* | 63(0.23)^*^ | SJCHGC09312 protein | *Schistosoma japonicum* | 32(0.2)^+‡^ | SJCHGC01679 protein | *Schistosoma japonicum* | 28(0.29) |
| 28 | AK010678 ribosomal protein S3 | *Schistosoma japonicum* | 58(0.21)^+^ | SJCHGC06675 protein | *Schistosoma japonicum* | 28(0.18)^+^ | SJCHGC01085 protein | *Schistosoma japonicum* | 28(0.29)^*^ |
| 29 | Predicted protein | *Nematostella vectensis* | 57(0.21)^*+^ | unknown | unknown | 28(0.18)^+‡^ | SJCHGC06340 protein | *Schistosoma japonicum* | 27(0.28)^‡^ |
| 30 | Unknown | unknown | 57(0.21)^*+^ | SJCHGC06166 protein | *Schistosoma japonicum* | 27(0.17)^+‡^ | SJCHGC06288 protein | *Schistosoma japonicum* | 26(0.27) |

Copy (%) indicates the percentage of a given EST in each ESTs (CSA: 27,070, CSM: 15,872, and CSE: 9,803) obtained from corresponding cDNA library.

The statistical analysis was performed as previously described (http://telethon.bio.unipd.it/bioinfo/IDEG6_form/index.html)

^*^ Significant differences of gene expression (*p*<0.01) between adult and egg.

^+^ Significant differences of gene expression (*p*<0.01) between adult and metacercaria.

^‡^ Significant differences of gene expression (*p*<0.01) between egg and metacercaria
