# Supplementary material for: Developmental Transcriptomic Features of the Carcinogenic Liver Fluke, Clonorchis sinensis
Source: PLoS Negl Trop Dis. 2011 Jun 28;5(6):e1208. doi: 10.1371/journal.pntd.0001208 (PMC3125140; doi:10.1371/journal.pntd.0001208)
Supplement: Table S2 — Putative parasitism-related genes of C. sinensis. (DOC) [file pntd.0001208.s004.doc]

**Table S2. Putative parasitism-related genes of *C. sinensis***

| **EST ID** | **Gene description** |
| --- | --- |
| CL4040Contig1 | Tyrosine kinase [*Monodelphis domestica*] |
| CL17Contig1 | Tubulin alpha-2 [*Fasciola hepatica*] |
| CL893Contig1 | Testis specific gene A2 [*Equus caballus*] |
| CSA07556 | Ribosomal protein L38 [*Homo sapiens*] |
| CSM14166 | Plakin repeat |
| CL2759Contig1 | Na^+^/Cl^-^ dependent neurotransmitter transporter-like protein [*Schistosoma mansoni*] |
| CL501Contig1 | mitoticphosphoprotein 90 [*Xenopuslaevis*] |
| CL299Contig2 | LIMPETin [*Schistosomamansoni*] |
| CL1031Contig1 | LDL receptor-like module |
| CL5070Contig1 | ALdehydedeHydrogenase family member (alh-1) [*Caenorhabditis elegans*] |
| CL1996Contig1 | Acylphosphatase |
| CL5580Contig1 | Unknown |
| CL4225Contig1 | Unknown |
| CL12Contig5 | Unknown |
| CL1005Contig1 | The predicted protein [*Nematostella vectensis*] |
| CL1535Contig1 | Unknown |
| CSA29823 | UPF0506 protein SJCHGC02965 precursor |
| CL2529Contig1 | CG12753 CG12753-PA [*Tribolium castaneum*] |
| CL4045Contig1 | Unknown |
| CL954Contig1 | Unknown |
| CL385Contig1 | Unknown |
| CL1527Contig1 | Unknown |
| CL613Contig1 | SJCHGC09379 protein [*Schistosoma japonicum*] |
