# Supplementary material for: Developmental Transcriptomic Features of the Carcinogenic Liver Fluke, Clonorchis sinensis
Source: PLoS Negl Trop Dis. 2011 Jun 28;5(6):e1208. doi: 10.1371/journal.pntd.0001208 (PMC3125140; doi:10.1371/journal.pntd.0001208)
Supplement: Table S3 — CsAEs of neuro-receptors and neurotransmitter producing enzymes according to C. sinensis developmental stage. (DOC) [file pntd.0001208.s005.doc]

**Table S3.** CsAEs of neuro-receptors and neurotransmitter producing enzymes according to *C. sinensis* developmental stage

|  |  | **No. of reads** | | |
| --- | --- | --- | --- | --- |
| **Category** | **Description** | **Adult** | **Metacercaria** | **Egg** |
| **Neuro-receptors** | Serotonin receptor-like planarian receptor 1 | 0 | 6 | 0 |
|  | Ionotropic glutamate receptor GLR-3 | 0 | 1 | 0 |
|  | GABA(A) receptor-associated protein-like 2 | 1 | 0 | 0 |
| N**euro-transmitter related proteins** | Glutaminase | 1 | 2 | 0 |
|  | Aromatic amino acid decarboxylase | 2 | 0 | 1 |
|  | DOPA-decarboxylase | 1 | 0 | 6 |
|  | Acetylcholinesterase | 0 | 3 | 0 |
|  | Serotonin transporter | 0 | 1 | 0 |
|  | Tryptophan hydroxylase | 0 | 2 | 0 |
