# Supplementary material for: Developmental Transcriptomic Features of the Carcinogenic Liver Fluke, Clonorchis sinensis
Source: PLoS Negl Trop Dis. 2011 Jun 28;5(6):e1208. doi: 10.1371/journal.pntd.0001208 (PMC3125140; doi:10.1371/journal.pntd.0001208)
Supplement: Table S4 — Putative drug targets of C. sinensis (DOC) [file pntd.0001208.s006.doc]

**Table S4. Putative drug targets of *C. sinensis***

| **EST ID** | **Adult** | **Metacercaria** | **Egg** | **Accession no.** | **Description** | **E-value** |
| --- | --- | --- | --- | --- | --- | --- |
| CSA02423 | 1 | 0 | 0 | ABU41051.1 | ADP-ribosylation-like factor 6 interacting protein | 3e-11 |
| CL164Contig1 | 31 | 0 | 0 | ACE06899.1 | Tetraspanins D76 and 18 | 2e-05 |
| CL1147Contig1 | 3 | 6 | 0 | XP_001659988.1 | Myelin proteolipid | 2e-16 |
| CSA06535 | 1 | 0 | 0 | NP_200294.1 | Dopamine beta-monooxygenase, N-terminal domain containing | 8e-06 |
| CL305Contig3 | 0 | 0 | 9 | NP_571737.1 | START domain containing 3 | 3e-11 |
